# Supported Catalytically Active Liquid Metal Solutions (SCALMS) for Propane Dehydrogenation–Intermetallic Phases and Liquid Alloys Studied by Pair Distribution Function Analysis and Density Functional Theory

**DOI:** 10.1002/advs.202511498

**Published:** 2025-09-09

**Authors:** Felix Egger, Andreas Mölkner, Julien Steffen, Nicola Taccardi, Narayanan Raman, Mirijam Zobel, Andreas Görling, Peter Wasserscheid, Marco Haumann

**Affiliations:** ^1^ Institut für Kristallographie RWTH Aachen University Jägerstraße 17–19 52066 Aachen Germany; ^2^ Department Chemie und Pharmazie Lehrstuhl für Theoretische Chemie Friedrich‐Alexander‐Universität Erlangen‐Nürnberg (FAU) Egerlandstr. 3 91058 Erlangen Germany; ^3^ Department Chemie‐ und Bioingenieurwesen Lehrstuhl für Chemische Reaktionstechnik (CRT) Friedrich‐Alexander‐Universität Erlangen‐Nürnberg (FAU) Egerlandstr. 3 91058 Erlangen Germany; ^4^ JCNS‐3: Neutron Analytics for Energy Research Forschungszentrum Jülich GmbH Wilhelm‐Johnen‐Straße 52428 Jülich Germany; ^5^ Erlangen National High Performance Computing Center (NHR@FAU) Martensstr. 1 91058 Erlangen Germany; ^6^ Helmholtz‐Institut Erlangen‐Nürnberg for Renewable Energy Forschungszentrum Jülich GmbH Cauerstraße 1 91058 Erlangen Germany; ^7^ Institute for a Sustainable Hydrogen Ecomony (INW) Wilhelm‐Johnen‐Straße 52428 Jülich Germany; ^8^ Research Centre for Synthesis and Catalysis Department of Chemistry University of Johannesburg P.O. Box 524 Auckland Park 2006 South Africa

**Keywords:** Alkane dehydrogenation, alloy composition, DFT, gallium, liquid alloy, pair distribution function, platinum

## Abstract

The supported catalytically active liquid metal solution (SCALMS) concept is based on catalytically active metals dissolved in a low‐melting‐point liquid metal matrix. These solid alloy particles, deposited over a high area support, transform into a liquid alloy under reaction conditions. In this work, GaPt SCALMS materials of varying composition are investigated and focus on the change in the alloy composition during preheating, the actual high temperature propane dehydrogenation at 823 K, and after cool‐down. X‐ray diffraction (XRD) is used and analyze the pair distribution function (PDF) combined with density functional theory (DFT) studies. Before catalysis, the Ga phase oxidizes to an amorphous β‐Ga_2_O_3_ phase which is likely removed during activation. Refinements for all sample data before catalysis confirm the presence of fcc‐Pt nanoparticles. After catalysis, the Pt nanoparticles convert to a GaPt_2_ alloy phase with high structural disorder, visible in the difference pair distribution function (dPDF). The machine‐learned force fields (ML‐FF) simulations suggest that GaPt_2_ forms at the Pt(111) interface with liquid Ga through gradual Ga intercalation into the Pt crystal structure. Simulations further indicate that both fcc‐Pt and GaPt_2_ are stable at temperatures up to 800 K if the Pt content of the surrounding liquid is high enough.

## Graphical abstract

Supported liquid alloys of gallium and platinum with different metal‐to‐metal ratios are used in propane dehydrogenation. The catalytic performance is correlated with spectroscopic insight gained from pair distribution function (PDF) studies combined with density functional theory (DFT).

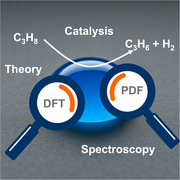

## 1 Introduction

**Figure 1:**
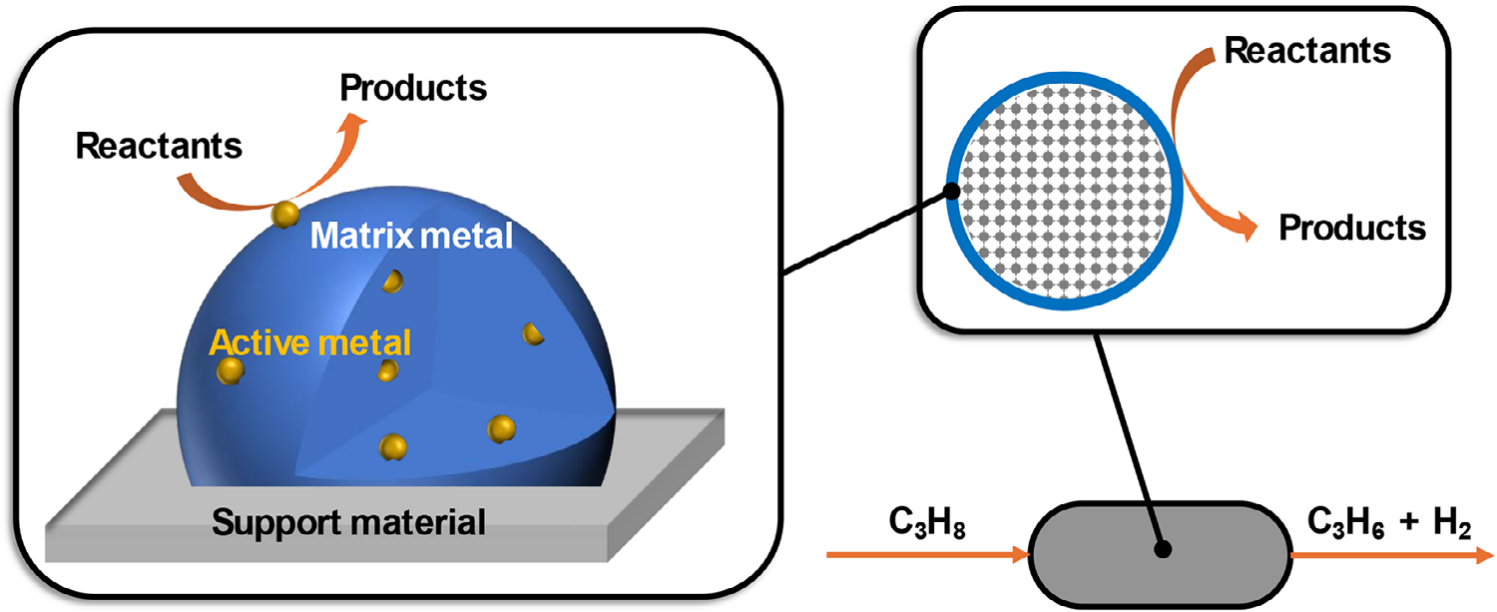
Schematic representation of the SCALMS material concept (left box) and the egg‐shell type alloy distribution of the outer surface area of the support (right box).

The development of more efficient catalysts for more benign chemical and energy processes is one of the key aspects for achieving future sustainability goals.^[^ ^1^ ^]^ Improved selectivity will reduce the amount of waste being produced and more robust catalysts will help to improve the overall energy efficiency of processes. Furthermore, with precious metals often being costly and from critical sources, their reduction or replacement is crucial.^[^ ^2^ ^]^ Several strategies have been developed over the past decades to reduce the precious metal inventory in chemical processes, including the use of nanoparticles or clusters down to single atom catalysis.^[^ ^3^, ^4^, ^5^ ^]^ Since the stabilization of these high energy clusters or atoms on support surfaces can constitute a problem for stability, we have developed a strategy for the dynamic stabilization of single atom catalysis based on liquid alloys, the so called supported catalytically active liquid metal solution (SCALMS) concept.^[^ ^6^ ^]^ SCALMS materials consists of small amounts of a catalytically active metal dissolved in a low melting point liquid metal matrix (see **Figure** **1**). These solid alloy particles, deposited over a high area support, transform into a liquid alloy under reaction operating conditions.^[^ ^7^ ^]^ It should be noted that SCALMS adds a promising engineering approach to the growing field of liquid metal catalysis, where bulk liquid alloy phases are present under reaction conditions.^[^ ^8^, ^9^, ^10^ ^]^

Various SCALMS catalysts have been tested successfully for different alkane dehydrogenation (T > 773 K, 0.1 MPa) and C‐C coupling reactions (T = 473 K, 0.6 MPa).^[^ ^11^ ^]^ The high catalytic activity of the SCALMS catalysts at reaction conditions is reasoned by the high dynamics at the gas/liquid alloy interface which generate very active single atom catalytic sites.^[^ ^12^ ^]^ These sites are protected from deactivation due to coking or sintering by the liquid nature of the alloy and the stabilizing effect of the Ga matrix. However, for high temperature processes like propane dehydrogenation at temperatures of ≈ 823 K, deactivation of SCALMS is observed. There is evidence that at the high temperatures > 773 K the Ga‐Pt droplets are mobile on the support surface.^[^ ^13^ ^]^ Note that Ga has a very poor wettability with most traditional catalyst support materials like SiO_2_, Al_2_O_3_ or SiC. To prevent the mobility, we developed new support materials based on supraparticles and suprabeads, where small cavities will entrap the droplets.^[^ ^14^ ^]^ While these concepts led to a reduction in deactivation degree, the deactivation could not be completely suppressed. During the movement on the support surface a segregation of the Ga‐Pt alloy into more Pt‐rich phases can occur, and these intermetallic phases solidify and become prone to classical coking.^[^ ^13^ ^]^ In this work, we aimed to study the possible formation of such intermetallic phases by means of X‐ray diffraction (XRD) and subsequent analysis of the pair distribution function (PDF) combined with density functional theory (DFT) studies.

Since liquid phases or nanocrystals, that do not exhibit long‐range periodicity and crystallinity, are crystallographically intractable with common approaches such as Rietveld refinement, high energy X‐ray total scattering with PDF analysis has become a common tool to investigate the short‐range order of nanoparticles, heterogeneous catalysts, liquids and alloys.^[^ ^15^, ^16^, ^17^, ^18^ ^]^ For such materials, crucial information about the atomic structure is contained in the diffuse scattering underneath and in between the Bragg peaks. The PDF is comparable to a histogram of the interatomic distances in the sample and is derived experimentally by performing a Fourier transformation of a powder diffraction pattern from the crystallographic reciprocal space to real space revealing interatomic distances.^[^ ^18^ ^]^ PDF experiments on heterogeneous catalysts are nowadays performed ex situ on dedicated laboratory PDF diffractometers, or in situ with neutrons or at synchrotron sources,^[^ ^19^ ^]^ reaching time resolutions on the sub‐second scale.

Throughout this article, the term PDF will be used for the G(r) function, which is also referred to as the reduced pair distribution function and oscillates around 0 at high r values.^[^ ^20^ ^]^ The term radial distribution function RDF will be used for the pair distribution function, which has been scaled by the average number density and is also often referred to as the pair density function g(r).^[^ ^16^ ^]^ At high r, the g(r) function oscillates ≈ 1.

To simulate the formation and stability of GaPt liquid and alloys, density‐functional theory (DFT) is the method of choice. It is able to describe the electronic interactions of the atoms with a high degree of accuracy, such that all relevant experimental phenomena can be reproduced.^[^ ^6^, ^21^, ^22^ ^]^ Unfortunately, due to its cubic scaling of the computational effort with the number of electrons, the usage of DFT is restricted to systems containing no more than some 100s of atoms, and to time scales of 10s to 100s of ps, which is insufficient for the real‐time sampling of the processes covered in this study. A viable alternative is to use a potential energy function that neglects the expensive treatment of electrons and replaces it by parameters optimized with the objective to reproduce the DFT ground truth as accurate as possible. In recent years, machine‐learned force fields (ML‐FFs) have emerged as powerful and versatile flavor of such optimized functions. In contrast to traditional approaches for the modeling of metals like the embedded atom model (EAM),^[^ ^23^, ^24^ ^]^ ML‐FFs do not rely on a set of physically motivated interatomic potential functions but are based solely on the comparison of atomic environments, defined by the translationally and rotationally invariant relative positions of the atomic cores. The crucial part for the setup of a ML‐FF is the parametrization. For this, a large number of structures and their respective DFT energies and atomic forces must be provided, and the ML‐FF learns the potential energy surface of the system from it. This procedure, however, is not trivial, since the ML‐FF does not contain physically‐motivated potential functions but is fully flexible, which allows for an arbitrary high quality of the description but also leads to instabilities and unphysical behavior if certain configurations of the system are not covered well enough by the reference data. The problem of collecting a sufficient and reasonable set of reference data is especially significant for liquid metal alloys and their surfaces, due to the many possible local configurations of the different elements. A straightforward learning strategy would be to run an ab‐initio molecular dynamics (AIMD) simulation, which needs to be long enough to cover all relevant configurations of the liquid metal alloy. Due to the high cost of DFT, this strategy is almost unfeasible for all but very simple systems like pure Ga. Instead, we use the on‐the‐fly learning algorithm, which was developed for the Vienna ab‐initio simulation package (VASP).^[^ ^25^, ^26^, ^27^ ^]^ It is based on the Gaussian process regression ML‐FF formalism and its ability to determine the uncertainty of any energy or force prediction.^[^ ^28^ ^]^ A VASP ML‐FF is learned ideally in one black‐box calculation. It starts as a usual AIMD calculation by a couple of DFT steps. The energies and gradients are used to learn an early version of the ML‐FF. In each MD step, the ML‐FF is used to predict the energy and forces of the next structure. If the uncertainty of this prediction is low enough, indicating a good coverage of the current configuration by the reference data collected so far, the ML‐FF prediction is used instead of DFT. If the uncertainty is above a certain threshold, DFT is used and its results added to the reference data. With this, the ML‐FF gradually learns larger parts of the relevant potential energy surfaces. Areas which are already known well are sampled by the much faster ML‐FF, restricting the expensive DFT calculations to unknown areas. With this, the learning is done efficiently and fully automated, although manual oversight and corrections are still advisable, especially for complicated systems. This on‐the‐fly method has been used by us for several studies of SCALMS and related systems in the past^[^ ^29^, ^30^, ^31^, ^32^, ^33^, ^34^, ^35^ ^]^ and thus is ideal for the simulation of GaPt liquid and crystalline alloys.

## 2 Experimental Section

### 2.1 SCALMS Material Synthesis

The SCALMS materials were prepared according to a published method.^[^ ^6^ ^]^ Briefly, 50.20 g of SiO_2_‐60 (35‐60 mesh) were impregnated with 130 mL of a 0.9 m solution of (Et)_3_NGaH_3_ in diethyl ether. The solvent was removed under vacuum at 253 K and the resulting solid was then heated up to 523 K and held at this temperature until no gas was produced. The resulting greyish material was then held under vacuum to remove any volatile followed by deposition of platinum via galvanic displacement. Here, the relevant amount of an aqueous H_2_PtCl_6_ stock solution (4.4 mg mL^−1^ Pt) was added to 5 g of Ga‐decorated SiO_2_ suspended in 30 mL of EtOH:H_2_O 5:1. After shaking for 2 h, the materials were filtered, washed in turn with water, ethanol, and acetone. The resulting solids were dried in an oven at 403 K overnight and the Ga and Pt loadings (in wt.%) of the prepared reference and SCALMS catalysts were determined by inductively coupled plasma atomic emission spectroscopy (ICP‐AES) using a Ciros CCD (Spectro Analytical Instruments GmbH, Germany). From the metal loading, the respective atomic ratios of Ga_x_Pt (x = 8, 14, 45, ad 117) were calculated. More details on synthesis and characterization can be found in the ESI.

### 2.2 SCALMS Catalyzed Propane Dehydrogenation

1.5 g of each SCALMS catalyst was loaded in a fixed bed quartz tubular reactor (see ESI for details). The reactor was heated to the set point of 823 K and 0.12 MPa at 10 K min^−1^ under an inert atmosphere of 100 mL_N_ min^−1^ argon (99.998% purity, Air Liquide). A pre‐treatment procedure for each SCALMS catalyst was carried out at 823 K for 3 h under a reductive atmosphere of 100 mL_N_ min^−1^ (20 vol.% H2, 80 vol.% Ar) followed by purging with 100 mL_N_ min^−1^ (100 vol. Ar) for 1 h. The reaction was started by supplying 8.9 mL_N_ min^−1^ propane (99.95% purity, Air Liquide) as feed gas diluted with 90.4 mL_N_ min^−1^ argon. Gases were dosed by mass flow controllers (MFC, Bronckhorst) to adjust a gas hourly space velocity (GHSV) of 3950 mL_gas_ g_Cat.bed_^−1^ h^−1^. The product gas mixture was analyzed using online gas chromatography (GC) on a Bruker 456 GC equipped with a GC‐Gaspro column (30 m x 0.320 mm) having a thermal conductivity detector (TCD) for detecting the light compounds (H_2_, Ar, He) and a flame ionization detector (FID) for detecting the C1‐C3 hydrocarbons. The conversion for propane, the selectivity for the desired product propene, the deactivation rate, and the catalyst productivity were calculated from calibrated peak area data (see ESI for details).

### 2.3 Pair Distribution Function Analysis

High energy X‐ray total scattering data for PDF analysis has been collected at PETRA III beamline P02.1 at DESY Hamburg (Germany) with an X‐ray energy of 59.78 keV (λ = 0.20735 Å). Data was collected for 900 s for each sample at room temperature over a *Q*‐range of 0.7 – 19.4 Å^−1^ using a Varex XRD 4343CT flat panel detector. Instrumental parameters for calibration of the detector distance to the sample as well as tilt and instrumental resolution parameters, *Q*_broad_ and *Q*_damp_, were determined by measuring a LaB_6_ (NIST660c) standard. Azimuthal integration of the 2D images was performed with the software package pyFai.^[^ ^35^ ^]^ Calculation of experimental PDFs was done with PDFgetX3 (https://doi.org/10.1107/S0021889813005190), structural refinements were conducted using Diffpy‐CMI.^[^ ^36^ ^]^ Measurements were performed for powder samples of all SCALMS catalysts in Kapton capillaries (1.1 mm OD) for the conditions as prepared, as well as after the propane dehydrogenation reaction. In addition, the pure silica gel support and the support loaded with pure Ga were measured as reference and to calculate difference PDFs (dPDF), by subtracting the total scattering data set of the pure support from the sample data prior to PDF calculation. In those dPDFs, only the atom‐atom correlations from the supported catalyst particles or correlations between the catalyst particle and support remain. PDF refinements were conducted up to 50 Å.

The PDF has been demonstrated as a tractable and intuitive approach for analyzing the structures of nanomaterials resulting in robust and quantitative structural solutions, where conventional XRD methods fail. Instrumental resolution parameters, Q_broad_ (0.002 Å^−1^) and Q_damp_ (0.03 Å^−1^) indicate high‐quality PDF data with good resolution. Of course, minor phase contributions and overlapping peaks (phase convolution) can always play a pivotal role in PDF analysis. This is for example the case for the first Ga–Ga and Pt–Pt distances, which is another reason why Ga was excluded from the refinements.

### 2.4 DFT and Machine Learning Simulations

All calculations presented in this paper were conducted using the VASP (Vienna Ab Initio Simulation Package) code, utilizing plane waves for valence electrons and the projector augmented wave (PAW) method for atomic cores.^[^ ^35^, ^36^, ^37^, ^38^, ^39^ ^]^ The Perdew, Burke, and Ernzerhof (PBE) exchange‐correlation functional was employed,^[^ ^40^ ^]^ with an electronic cutoff of 250 eV and a convergence criterion for electronic minimizations of 10^−7 ^eV. In geometry optimizations of intermetallic compounds unit cells and atomic positions were relaxed to a maximum force component criterion of 5 × 10^−3^ eV/Å. First‐order Methfessel‐Paxton smearing with a width of 0.2 eV was used.^[^ ^41^ ^]^ Dispersion interactions were considered using Grimme's D3 method.^[^ ^42^, ^43^ ^]^ Two VASP ML‐FFs were parametrized in the line with study.^[^ ^26^, ^27^, ^44^ ^]^

The on‐the‐fly learning of the ML‐FF for the dissolution of Pt atoms from a Pt(111) surface into liquid Ga involved a unit cell with a Pt(111) surface (4 layers, 120 Pt atoms) and 150 Ga atoms arranged on a cubic grid. The unit cell was orthorhombic with dimensions a = 13.91 Å, b = 14.46 Å, and c = 42 Å, and was sampled using a 2 × 2 × 1 k‐point mesh. The on‐the‐fly learning simulation ran for 500000 steps at 1500 K, using the Nose‐Hoover thermostat,^[^ ^45^, ^46^ ^]^ with a limit of 6500 local reference configurations. Cutoffs for radial and angular descriptors were set to 8 Å, and 8 and 5 Å for refitting the fast ML‐FF without Bayesian error estimation.

The second ML‐FF, used for the dissolution of GaPt_2_ and Pt crystals in liquid GaPt with varying Pt concentrations, was built up from different independent on‐the‐fly learnings, which reference data were combined with our new mlff_select program, being part of the utils4VASP repository on github [https://github.com/Trebonius91/utils4VASP, paper will be submitted soon]. The first piece of reference data is that of the first ML‐FF. Further, a system consisted of 100 Ga and 100 Pt atoms, randomly placed on a 5 × 5 × 8 grid, underwent linear heating from 300 to 1000 K over 1000000 steps. The number of reference structures and configurations for this simulation was raised to 5500. Finaly, different GaPt intermetallic phases (including Ga_5_Pt, Ga_7_Pt_3_, Ga_3_Pt_2_, GaPt, and GaPt_2_) were learned on the fly, with Ga_2_Pt not explicitly trained since the trained force field was capable of crystallizing Ga_2_Pt structures. Details for these training runs can be found in the Supporting Information.

The Pt(111) surface dissolution simulation paired multiple cells of different sizes containing 5 layers of Pt(111) with liquid Ga on top. Temperatures of 400, 500, 600, 700 K, and 800 K were simulated with 24 million MD steps, each. Stability evaluations of GaPt_2_ and Pt nanoparticles in varying concentration liquid SCALMS were executed at 300 K to 900 K using the second ML‐FF, with cubic boxes containing 8000 atoms and six different Pt concentrations. Each setup was equilibrated for 50000 MD steps using the Parrinello‐Rahman barostat, inserted nanoparticles were supercells of intermetallic phases. The modify_poscar.py script from the utils4VASP repository was employed to place nanoparticles in the liquid matrix, with atoms less than 2.3 Å from the nanoparticles removed. Each configuration was sampled for 300000 MD steps. For reference radial distribution functions, a Ga_2_Pt supercell with 512 Ga and 1024 Pt atoms was simulated for 50000 steps at 300 K.

## 3 Results and Discussion

### 3.1 Propane Dehydrogenation

**Figure 2:**
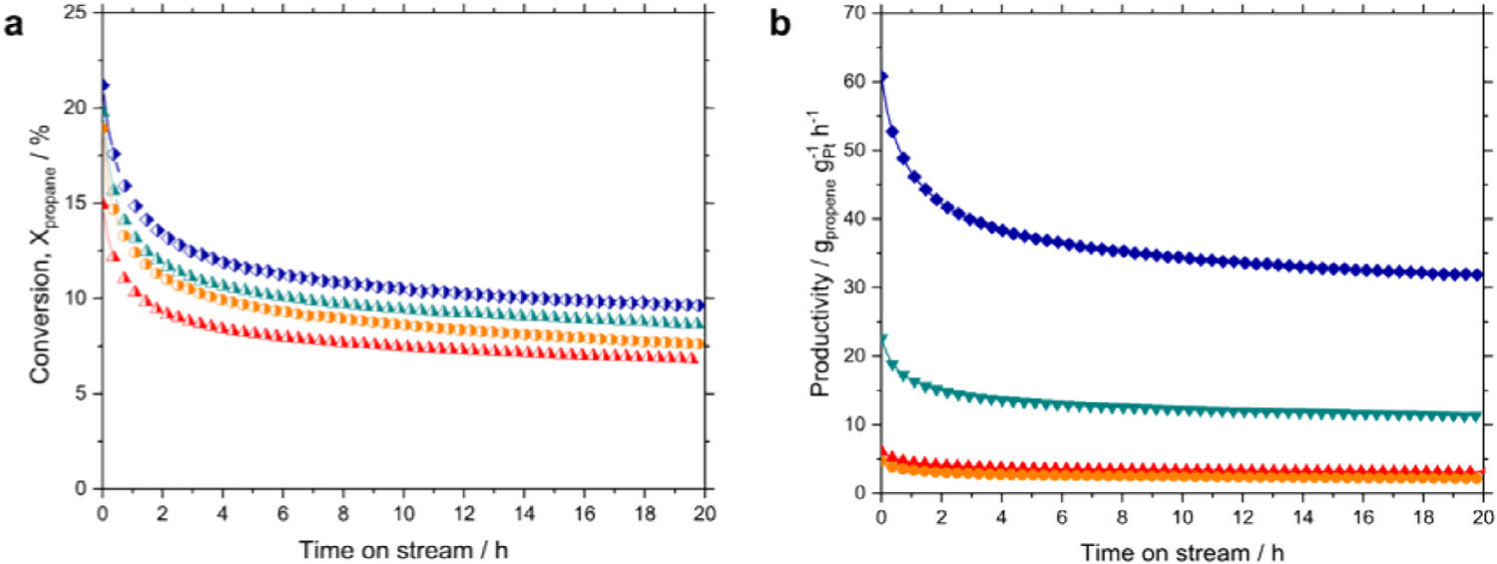
Continuous gas‐phase propane dehydrogenation using GaPt SCALMS catalyst with different atomic ratios Ga_8_Pt (orange), Ga_14_Pt (red), Ga_45_Pt (green) and Ga_117_Pt (blue). Reaction conditions: 1.2 g catalyst, He flow 89 mL_N_ min^−1^, C_3_H_8_ flow 8.9 mL_N_ min^−1^, GHSV 4900 ml_gas_ g_Cat_._bed_^−1^ h^−1^.

The comparison of four GaPt‐SCALMS catalysts in the continuous gas‐phase propane dehydrogenation at 773 K and 0.12 MPa total pressure is shown in **Figure** **2**. The catalysts differed in the Ga to Pt atomic ratio (Ga_x_Pt with x = 8, 14, 45, 117) while all other parameters were kept constant. We wanted to investigate at least two systems that do not form a fully liquid system under reaction conditions, hence the values of eight and 14 were selected. The value of 45 is close to the liquidus line while the value of 117 definitely yields fully liquid SCALMS‐system under reaction conditions.^[^ ^47^ ^]^

All catalysts started at high and comparable initial conversion levels around 23% but lost activity within the first 5 h of time on stream. During the next 15 h, the deactivation was much less pronounced but still present. Highest conversion was achieved with the Ga_117_Pt catalyst, containing the lowest amount of precious metal, while the lowest conversion level after 20 h was observed for Ga_8_Pt, the latter having the highest metal content. When calculating the productivity for propene formation, the differences became more pronounced as shown in Figure 2b. The final productivity level for Ga_117_Pt was 35 g_propene_ g_Pt_^−1^ h^−1^, almost ten times higher than for Ga_14_Pt and Ga_8_Pt. This supports the fact that the SCALMS concept allows for a more efficient use of the precious active metal and is in good agreement with our previous findings on varying the active metal loading for Pt and Rh in SCALMS‐catalyzed PDH.^[^ ^7^, ^8^, ^12^ ^]^ Both Ga_14_Pt and Ga_8_Pt should not liquefy under reaction conditions, according to the Ga‐Pt phase diagram.^[^ ^47^ ^]^ Therefore, part of the Pt is not available for the catalysis as it is stuck in the bulk of the solid intermetallic phases formed with Ga. On the other hand, the Ga_45_Pt should be close to the liquidus line at 773 K and both liquid alloy as well as intermetallics should be present. Finally, only the Ga_117_Pt should be fully liquid under the propane dehydrogenation conditions, thus allowing for high mobility of the Pt atoms close to the surface of the liquid alloy droplets. When plotting the initial and final values of conversion and productivity over the content of active Pt metal, only a small dependency is observed for conversion, while a strongly exponential decay is observed for productivity (see Figure S2, Supporting Information). The strong increase in productivity at higher gallium content again exemplifies the importance of the alloy being fully liquid to maximize the utilization of the precious Pt metal. It should however be noted that the accuracy of the determined phase diagram in the Pt‐lean region is low, with strong deviations from different literature data. To shed light on the observed correlation between phase behavior and catalytic performances, we carried out difference pair distribution function (dPDF) and density functional theory (DFT) studies.

### 3.2 PDF Analysis of Ga‐Pt SCALMS

**Figure 3:**
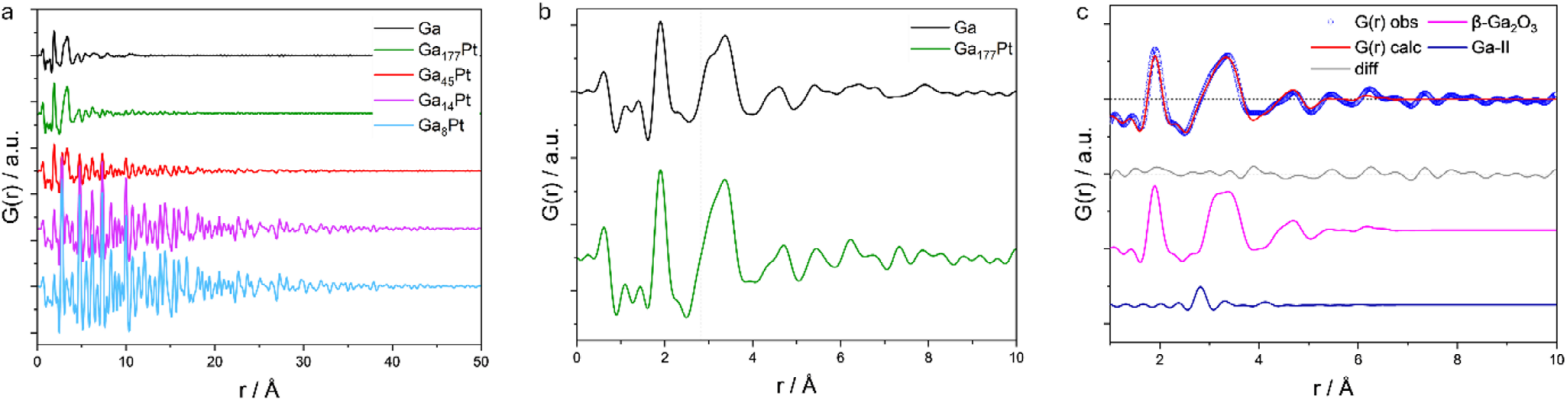
a) Experimental dPDFs of the four different SCALMS catalysts and the pure Ga phase before catalysis after subtraction of the SiO_2_ support signal. b) zoomed in version of the pure Ga and Ga_177_Pt catalyst with a red vertical dashed line at 2.8 Å for highlighting the shoulder in the Ga_177_Pt signal, which might stem from a possible Ga‐Ga next neighboring distance. c) Refinement of the Ga_177_Pt dPDF with the two phases β‐Ga_2_O_3_ and Ga‐II.

The experimental difference pair distribution function (dPDF) data sets of the different SCALMS samples, after subtraction of the contribution from the SiO_2_ support, show distinctly different characteristics, see **Figure** **3**a. The dPDF of Ga_177_Pt shows a high similarity to the dPDF of pure Ga supported on SiO_2_ (after subtraction of the SiO_2_ signal). From the r range, over which the PDF peaks extend before the PDF signal decays to zero, we can estimate the range of short‐range order in case of disordered materials or the crystalline domain size in case of crystalline materials.

The dPDFs of pure Ga and Ga_177_Pt only show significant peaks up to about 15–20 Å, reflecting only short‐range order as typical for glasses, liquids and amorphous phases. The dPDF of Ga_177_Pt features three significant interatomic distances, see Figure 3b, with the nearest‐neighbor distance at ≈1.91 Å followed by distances at 3.08 and 3.38 Å, which are combined in a broad peak. The presence of such broad peaks in the PDF is typically associated with increased variability in bond lengths and angles as commonly observed in amorphous materials. All PDF data were collected at room temperature, at which nanosized metal particles in SCALMS catalysts are known to be present in liquid state. For instance, some of us showed via SEM images, that supported Pd‐containing Ga‐rich phases feature liquid Ga droplets with a scale of hundreds of nanometers up to the micrometer range on top of the support even at room temperature.^[^ ^6^ ^]^

The nearest‐neighbor distance in metallic α‐Ga, which is also known as Ga‐I, is 2.44 Å.^[^ ^48^ ^]^ On the contrary, in liquid Ga, whose short‐range order closely correlates with that of the high‐pressure crystalline Ga‐II and Ga‐III phases according to,^[^ ^49^, ^50^ ^]^ the first interatomic distance occurs at 2.78 and 2.81 Å, respectively.^[^ ^51^ ^]^ This implies that another structure must account for the first observed distance at ≈1.91 Å. We attribute this distance to an amorphous β‐Ga_2_O_3_ phase, which exhibits a Ga‐O distance of 1.83 Å for tetrahedral and 2.00 Å for octahedral coordination.^[^ ^52^ ^]^ Since both distances are close to each other, they merge into a single peak in the observed PDF at 1.91 Å. This indicates that the Ga phase must have undergone oxidation to a certain extent.

The dPDF refinement of the Ga_177_Pt catalyst with a β‐Ga_2_O_3_ phase (ICSD 34243) up to a range of 10 Å results in a goodness‐of‐fit value R_w_ of 0.25. The R_w_ value is a quantitative measure for the goodness of the fit of a theoretical model structure to the experimental data (similar to the meaning of goodness‐of‐fit values in powder X‐ray diffraction refinements).^[^ ^53^ ^]^ To determine the crystalline domain size of the β‐Ga_2_O_3_ phase, we include a corresponding fit parameter into the PDF refinement, which models the decay of the PDF peak intensities with increasing r. The refined domain size for β‐Ga_2_O_3_ in the Ga_177_Pt data is ≈7 Å and hence smaller than one unit cell in c direction with 12.23 Å. This further supports the hypothesis that the gallium oxide phase is amorphous. The formation of a gallium oxide phase at the surface of liquid alloy particles has been postulated for various Ga‐based alloys).^[^ ^6^, ^8^ ^]^ The oxide layer has been proposed to be amorphous,^[^ ^54^ ^]^ which suggests that the formation of an amorphous phase is thermodynamically favored on a liquid surface, supporting the idea of a liquid Ga phase before oxidation. The surface oxidation of Pt/Ga alloys in particular, has already been investigated,^[^ ^55^ ^]^ where the formation of a gallium oxide layer on the liquid Pt/Ga surface has been analyzed by near ambient pressure XPS analysis, using different oxygen partial pressures and temperatures from 300 up to 550 K. The growth of the oxide film was limited by mass transport and its thickness does not reach a plateau even after 280 min of exposure to 1 hPa of O_2_ at 450 K. Treatment at 300 K and an oxygen partial pressure of 1 hPa lead to an oxide layer thickness of ≈ 15 Å after 120 min. Removing these surface layers before catalysis in an activation process allows to reach the full catalytically active SCALMS state of the system.^[^ ^8d^ ^]^ Furthermore, Grabau, M. et al. reported on a surface enrichment of Pt in β‐Ga_2_O_3_ of up to 3.6 at.%, which cannot be confirmed or refuted based on the PDF data.^[^ ^55^ ^]^

As the samples for this study were synthesized and handled at ambient conditions, the formation of only a thin oxide layer at the surface becomes less plausible. In addition, the absence of an extended crystalline structure of gallium oxide in the dPDF, together with the absence of any significant residual signal corresponding to metallic or liquid Ga, strongly suggests the formation of a bulk amorphous gallium oxide phase for all samples. Furthermore, the sample preparation for PDF analysis, which included grinding the sample to a fine powder, increases the surface area for Ga to oxidize, enhancing this effect and biasing our observations toward the formation of an amorphous oxide phase. Consequently, it appears that oxidation proceeds much more extensively throughout this study than postulated by most previous articles. However, in another study with similar sample preparation, we were able to show the complete reduction of β‐Ga_2_O_3_ to liquid Ga via Raman spectroscopy.^[^ ^56^ ^]^

Evidence for a coexisting liquid Ga phase in the PDF can only be suspected based on a distance at 2.8 Å, in the form of a shoulder on the above mentioned broad peak spanning the range of 2.6–3.7 Å (Figure 3b), that results from the second and third interatomic distance of β‐Ga_2_O_3_, making the distinct identification of the 2.8 Å peak challenging. By adding a Ga‐II structure (ICSD 12173) to the refinement, the goodness‐of‐fit improves to 0.21 (Figure 3c). This result is consistent with the hypothesis in literature that the Ga droplets can be described with a core‐shell model consisting of a Ga core surrounded by a gallium oxide shell. Yet, given that we introduce an additional fit parameter by adding the Ga phase to the PDF refinement, the improvement in goodness‐of‐fit R_w_ should not be over‐interpreted considering the comparably small scale of this Ga phase. Like the addition of a Ga‐II phase, the addition of a Pt_fcc_ phase during the refinement of Ga_177_Pt data reduces the R_w_ value to 0.18. Since the particle size and scale of the phase contribution align closely with the results for the other samples, we regard the formation of Pt nanoparticles in this sample as likely, too. An increase in the long‐range order and thus crystallinity in the different SCALMS samples can be observed for increasing Pt content. The dPDF of Ga_45_Pt extends to higher r‐values than that of Ga_177_Pt, with several well‐defined peaks in the PDF for Ga_45_Pt.

For Ga_14_Pt and Ga_8_Pt, the dPDF signals extend up to 40 Å. Refinements of the dPDFs confirm the existence of Pt_fcc_ nanoparticles with increasing particle diameter for increasing Pt content: 2.5 nm for Ga_177_Pt and for Ga_45_Pt, 4.0 nm for Ga_14_Pt and Ga_8_Pt. Simultaneously to the increasing size of Pt nanoparticles, refinements show a decrease in their R_w_ values, which can be correlated to the increasing Pt content. Refinements of the Ga_8_Pt and Ga_14_Pt catalysts with a β‐Ga_2_O_3_ and a Pt_fcc_ structure (ICSD 243 678) result in an R_W_ of 0.13 and 0.16 respectively, indicating the formation of pure Pt nanoparticles without any detectable formation of a Pt‐Ga alloy, prior to catalysis. Although the signals arising from the Pt_fcc_ phase dominate the pattern because of the strong X‐ray scattering power of heavy elements, the PDF peaks for Ga‐Ga, Ga‐O and O‐O distances of much weaker intensities can be reliably identified in the PDF. The same refinements, as for Ga_8_Pt and Ga_14_Pt, conducted for Ga_45_Pt resulted in a slightly higher R_w_ value of 0.26.

In the samples investigated here, the greatest discrepancies between the calculated PDFs and the observed ones are accumulating at the low r range for all refinements (Figure S3, Supporting Information). This can be explained by the β‐Ga_2_O_3_ phase being amorphous, while we use a crystalline phase of very small domain size to model it. Another explanation could be size‐related effects, as small particles exhibit more lattice deviations due to surface and structural defects.^[^ ^53^ ^]^ As a result, increasing platinum particle size improves the overall fit quality.

**Figure 4:**
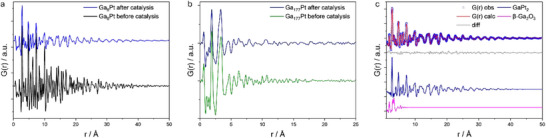
a) Experimental dPDFs of the Ga_8_Pt SCALM catalyst before and performing catalysis. b) Experimental dPDFs of the Ga_177_Pt SCALM catalyst before and performing catalysis. c) Refinement of the Ga_8_Pt dPDF after catalysis with the two phases β‐Ga_2_O_3_ and GaPt_2_.

The post‐catalysis PDF results show a significant deviation from the initial state, indicating a substantial structural evolution driven by the catalytic process. While this effect can be observed for all samples, it is most pronounced for the Ga_8_Pt (**Figure** **4**a) and Ga_14_Pt, which have the highest Pt content. Although their PDFs show significant peak broadening for distances above 8 Å, the local structure from 0 to 8 Å is similar to the one before catalysis. Peaks that we attributed to the Pt phase differ only in their relative intensity, while their position remains unchanged. Overall, the dPDF appears to reflect a less crystalline structure after catalysis, as indicated by broader PDF peaks and lower peak intensities. Peak broadening at higher r‐values in the PDF is usually accounted for by adjusting the atomic displacement parameters (ADPs), called B_iso_. The treatment of thermal motion in the PDF is based on models that are similar to the Debye‐Waller factors commonly used in crystallography to describe how thermal motion affects scattering intensity. Peak broadening in the PDF can be a result of both uncorrelated thermal motion of atoms or static displacements of atoms away from their ideal position within the lattice (ISBN: 0 08 042698 0). Since the samples were all investigated at room temperature, we allocate differences in the ADP values of different samples rather to varying structural disorder rather than a temperature effect.

The features of the PDF that can be observed in the post‐catalysis samples can be best explained by the complete conversion of Pt_fcc_ nanoparticles into a GaPt_2_ alloy phase (ICSD 103919) (often referred to as Pt_2_Ga in the literature), which must have formed either during catalytic conditions or during the cooldown process. The crystal structure of GaPt_2_ exhibits a nearest‐neighbor Pt‐Pt distance of 2.73 Å, which is similar to the Pt‐Pt distance in Pt_fcc_ (2.77 Å). As a result, this phase would produce a peak at nearly the same position. However, the peak intensities will differ due to variations in the coordination spheres arising from the distinct crystal structures of GaPt_2_ (*Pmma)* and Pt ($Fm\bar{3}m)$, as well as the incorporation of Ga into the GaPt_2_ alloy. Since Ga has a lower scattering power compared to Pt, it further influences the intensity of the observed peaks.

The formation of the GaPt_2_ structure can be observed for all samples after catalytic cycling but the Ga_117_Pt catalyst, where changes appear to be minor (Figure 4b). Refinements for the Ga_8_Pt (Figure 4c) and Ga_14_Pt catalyst after 20 h propane dehydrogenation, only using a β‐Ga_2_O_3_ and GaPt_2_ phase, result in a R_w_ of 0.17 and 0.19, respectively, and a domain size of 5 nm in both cases. The same refinement for the Ga_45_Pt catalyst results in a R_w_ of 0.30 with a size of 3 nm. The peak broadening at higher r‐values is accounted for by adjusting the model ADP values.

Refinements for the Ga_177_Pt catalyst using a GaPt_2_ alloy phase were unsuccessful, which may be due to the low Pt content. The low Pt content could result in a very small amount of a highly disordered structure, making it difficult for the PDF refinement to structurally refine this phase. Refinements for the Ga_177_Pt sample, performed only with the β‐Ga_2_O_3_ structure result in an R_w_ of 0.37. Neither adding the GaPt_2_ nor a Pt_fcc_ structure improved the quality of the fit, speaking for a homogeneous dispersion of metallic Pt or Pt‐Ga alloy particles in the Ga_177_Pt SCALMS catalyst after the propane dehydrogenation reaction.

The formation of a Pt‐Ga alloy could be one of the reasons for the poorer performance for the catalysts Ga_8_Pt, Ga_14_Pt and Ga_45_Pt, providing better evidence for the proposed influence of the Pt‐Ga alloy on the deactivation of the catalyst, as suggested by the catalysis data.

Peaks associated with the β‐Ga_2_O_3_ phase appear unaffected by the catalytic process and unchanged compared to pre‐catalysis samples, which implies the re‐formation of the phase after catalysis under ambient conditions.

### 3.3 DFT and ML Simulation of Ga‐Pt Phase Behavior

**Figure 5:**
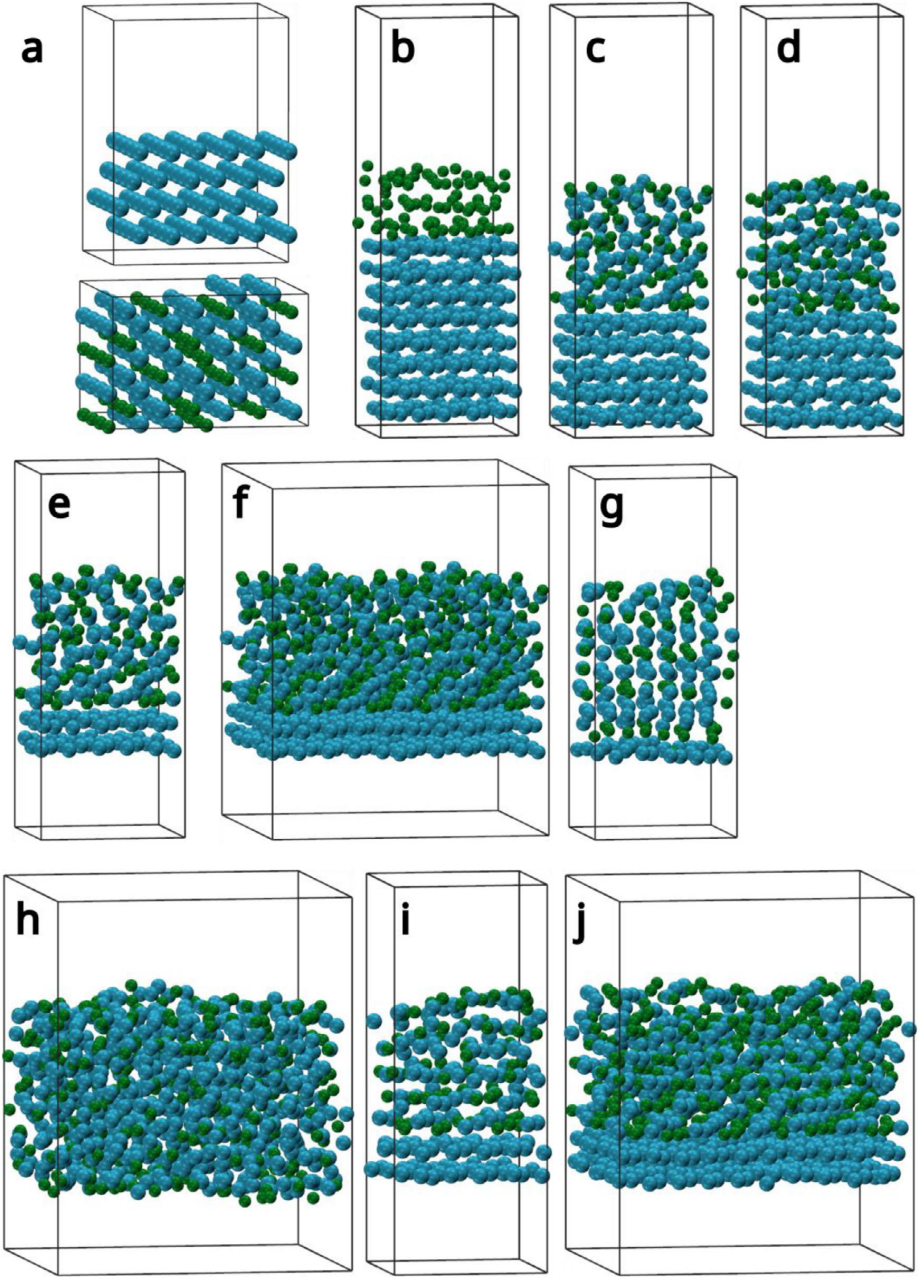
Selected screenshots from the MD trajectories simulating the dissolution of a Pt(111) surface in contact to liquid Ga, at different temperatures and with different unit cell sizes, Ga atoms are presented in green and Pt atoms in blue. a) Supercells of Pt metal and the GaPt_2_ intermetallic compound. b) Initial structure of the 8 Pt(111) layers in contact with liquid Ga. c) The same cell after 16 ns simulation time at 800 K. d) The same cell after another 32 ns simulation time at 800 K. e) The cell in (b) but with the lowest three Pt(111) layers removed. f) A 2 × 2 supercell of (e). g) Cell € after 40 ns simulation time at 800 K. h) Cell (f) after 40 ns simulation time at 800 K. i) Cell € after 40 ns simulation time at 600 K. j) Cell (f) after 40 ns simulation time at 600 K.

**Figure 6:**
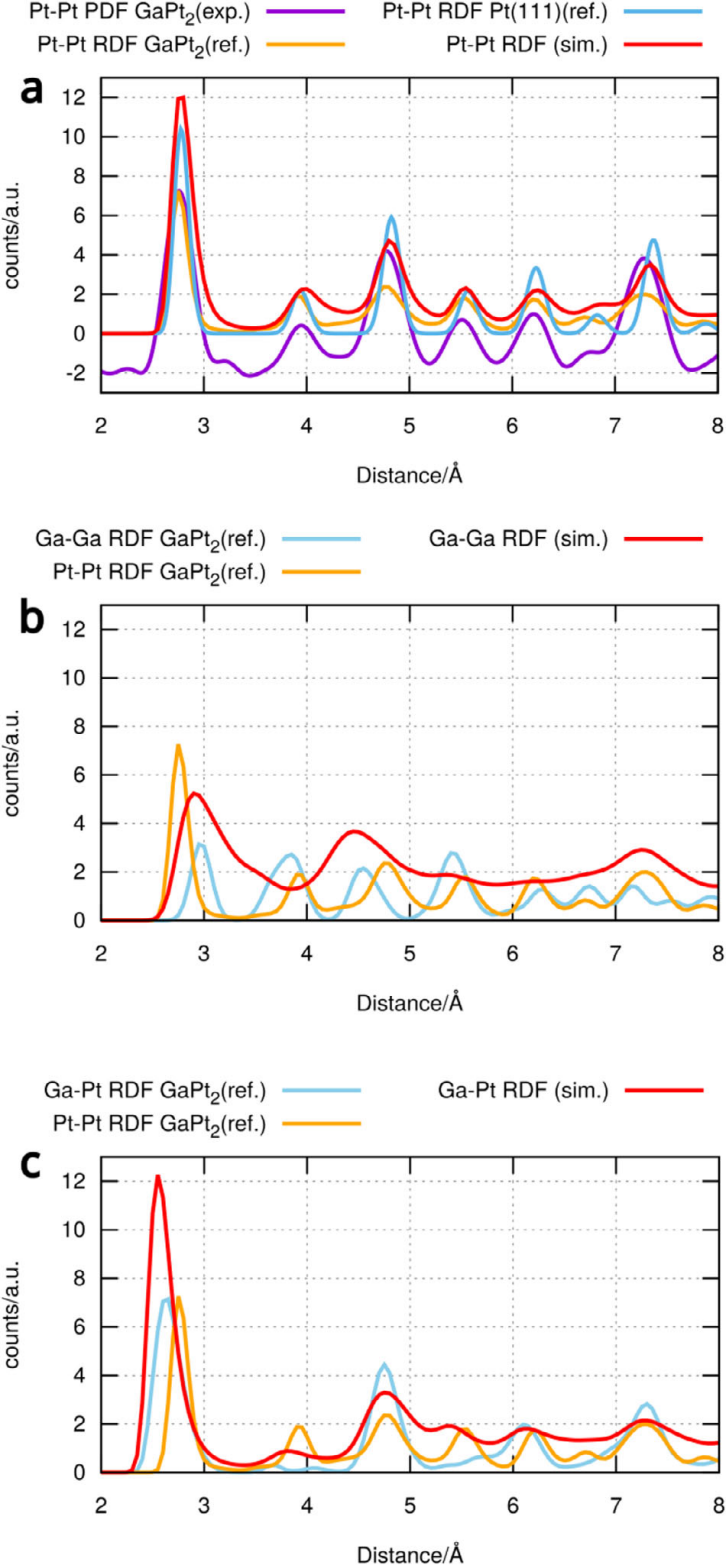
Comparison of experimental Pt‐Pt RDFs obtained from the dissolution of Pt rafts in liquid Ga with the reference Pt‐Pt RDF of GaPt2 and Pt(111) and the RDFs obtained at the end of the dissolution simulations of the Pt(111) surface slab in contact with liquid Ga. a) Comparison of the Pt‐Pt PDFs and the reference Pt‐Pt RDF for Pt(111) and GaPt_2_ with the simulated Pt‐Pt RDF from the 2 × 2 cell after 8 ns at 300 K. b) Comparison of the calculated reference Ga‐Ga and Pt‐Pt RDF of crystalline GaPt_2_ with the simulated Ga‐Ga RDF from the 2 × 2 cell after 8 ns at 300 K. c) Comparison of the reference Ga‐Pt and Pt‐Pt RDFs of crystalline GaPt2 with the simulated Ga‐Pt RDF from the 2 × 2 cell after 8 ns at 300 K.

Looking at the crystal structure of GaPt_2_ (shown in **Figure** **5**a) it is quite similar to fcc Pt, especially when looking at the Pt(111) surface facet. This observation is further strengthened upon comparison of the radial distribution functions in **Figure** **6**. Due to this a simulation cell consisting of 8 layers of Pt(111) with 3 layers of melted Ga on top (Figure 5b) was used to simulate the intrusion of Ga into Pt. This resulted in a concentration of 76% Pt and 24% Ga. The simulation was run for 8000000 steps of 2 fs at a constant temperature of 800 K, which would be totally out of reach for a direct DFT simulation. In this time the Ga was able to intrude into the first 3 Layers of the Pt surface (Figure 5c). While the top of GaPt phase showed liquid behavior, the two layers at the Pt interface showed the same structure as Pt(111). A consecutive simulation for 16000000 steps at 800 K showed no further Ga intrusion into the Pt surface (Figure 5d). To further investigate the structure of the GaPt phase on Pt(111), the simulation cell was modified. In order to reduce the influence of the Pt(111) phase on the RDFs averaged over all atoms in the simulation cell, the lowest three layers of the Pt surface were removed (Figure 5e). This cell and a cell multiplied in the x and y direction (2 × 2) (Figure 5f) was simulated at 400 to 800 K for 2000000 steps each temperature. In the case of the small cell at 800 K (Figure 5g) Ga intruded further into the last two remaining layers of Pt and formed one GaPt phase, similar to GaPt_2_.

In the case of the 2 × 2 cell the system showed liquid behavior at 800 K (Figure 5h) and formed no structures. At 700 K and below the two Pt layers were retained. Furthermore, at 700 and 600 K the whole system showed the Pt 111 structure (Figure 5i,j). At 500 and 400 K the initial liquid GaPt phase started to crystallize but the low mobility of the atoms at these temperatures prevented a full crystallization. The final structure of the multiplied cell at 600 K (Figure 5ij) was simulated at 300 K for 4000000 steps and the trajectory was used to obtain the radial distribution functions shown in Figure 6.

Figure 6a shows the similarity between the Pt‐Pt radial distribution functions of Pt(111) and GaPt_2_. Furthermore, the similarity with the pair distribution functions is shown. The simulated RDF at 300 K of the 2 × 2 cell show the same peaks as the references which indicates that the formed structure is similar to GaPt_2_. When comparing the reference Ga‐Ga and Pt‐Pt RDF of GaPt_2_ in Figure 6b, it is visible that Ga and Pt occupy similar positions in the crystal. The Ga‐Ga RDF from simulations with the 2 × 2 cell show three peaks at 3, 4.5, and 7.3 Å, which fits to the GaPt_2_ structure. Furthermore, it shows small peaks at the peak positions between 5 and 7 Å. The big peak at 3.8 Å present in the Ga‐Ga and Pt‐Pt RDFs of GaPt_2_ is missing which shows that the exact structure is not formed. However, a similarity to GaPt_2_ can be seen in the RDF data. Comparing the reference Pt‐Pt and Ga‐Pt RDFs (Figure 6c) of GaPt_2_ shows that Ga occupies the first, third and fifth shell positions around Pt. This structure is reproduced by the simulation, but it also shows Ga in the second and fourth layer around Pt which should be occupied by Pt. This indicates that the underlying structure of the simulated system is similar to GaPt_2_ but the finer structure and especially the position of Ga in the crystal is not fully reflected.

In the next step, the stability of GaPt_2_ and Pt crystals in liquid GaPt solutions was investigated at different temperatures. The first objective is to determine if our ML‐FFs are able at all to describe the GaPt_2_ intermetallic phase, the second objective is to obtain a more quantitative trend of the stability of the phase depending on the Pt concentration around it and the temperature. For this, the training set of the first ML‐FF was extended to include explicit training data of several important GaPt intermetallic phases and liquid GaPt, to improve the stability and reliability of the force field with respect to the whole relevant GaPt phase diagram, leading to the second ML‐FF. See in the experimental part for further details.

**Figure 7:**
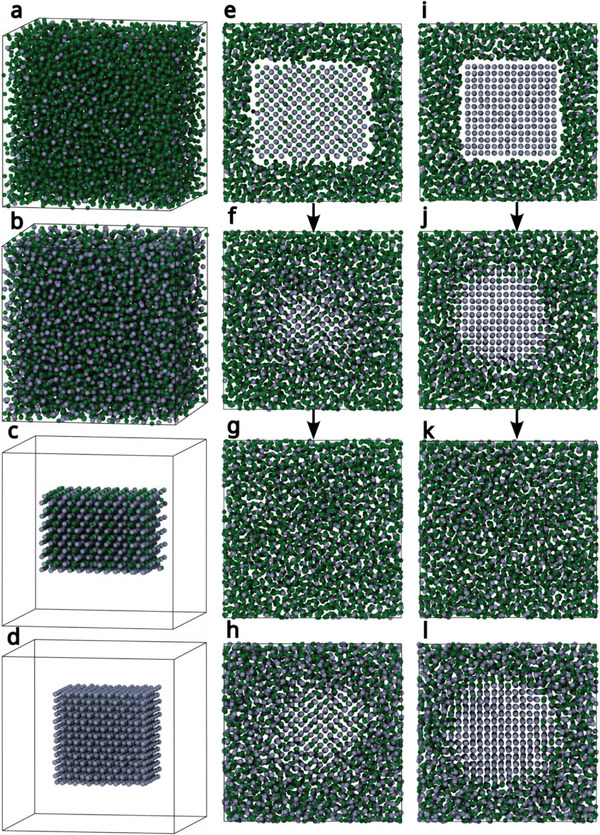
Screenshots of the placement and solvation of GaPt_2_ and Pt nanoparticles into liquid GaPt of different Pt concentrations (16.6% and 50%), Ga atoms are presented in green and Pt atoms are in blue. a) Preequilibrated box containing liquid GaPt with 16.6% Pt. b) Prequilibrated box containing liquid GaPt with 50% Pt. c) The GaPt_2_ nanoparticle to be inserted into the liquids. d) The Pt nanoparticle to be inserted into the liquids. e) The initial structure of GaPt_2_ inserted into liquid GaPt (16.6% Pt). f) The same cell as (e) after 1.5 ns simulation time at 400 K. g) The same cell as (e) after 1.5 ns simulation time at 700 K. h) GaPt2 inserted into liquid GaPt (50% Pt) after 1.5 ns simulation time at 700 K. i) The initial structure of Pt inserted into liquid GaPt (16.6% Pt). j) The same cell as (i) after 1.5 ns simulation time at 400 K. k) The same cell as (i) after 1.5 ns simulation time at 700 K. l) Pt inserted into liquid GaPt (50% Pt) after 1.5 ns simulation time at 700 K.

Some example screenshots of the performed simulations are shown in **Figure** **7**. Parts (a) to (d) show how the initial structures were set up. Preequilibrated liquid GaPt phases containing 16.6% and 50% of Pt atoms ((a) and (b)) at different temperatures constitute the solvents in which GaPt_2_ and Pt crystals ((c) and (d)) are placed with the modify_poscar.py script of the utils4VASP repository. The resulting initial structures in the liquid GaPt alloy with 16.6% Pt content are shown in (e) and (i). To accentuate the visibility of the crystals placed in the middle of the cell, the front part of the cell was cut away. In (f), the same structure as in (e) is shown after 1.5 ns sampling at 400 K. The crystalline area shrunk and rotated somewhat but is still clearly visible and seems to be in an equilibrium with the region of increased Pt content originated from the dissolved crystal around it. At 700 K, however, the crystal got fully dissolved, resulting in a homogeneous GaPt liquid of increased Pt content (g). If the surrounding liquid already contains 50% Pt from the beginning (h), however the GaPt_2_ crystal remains present also at 700 K and is even somewhat larger than the one in the liquid alloy with 16.6% at 400 K (f). A similar trend can be seen for the Pt crystal ((i) – (l)). Its edges got rounded up at 400 K in 16.6% Pt solution (j), but most of it remains, whereas it is fully dissolved at 700 K (k). If the GaPt solution contains 50% Pt, however, the Pt crystal again remains fully stable (l) and even seem to have grown somewhat at the outer edges of the resulting sphere. It can thus be followed that higher Pt concentrations of the surrounding liquid favor the stability of both GaPt_2_ and Pt particles at higher temperatures, whereas the GaPt_2_ and Pt particles are only stable in alloys with lower Pt content if the temperature is lower.

**Figure 8:**
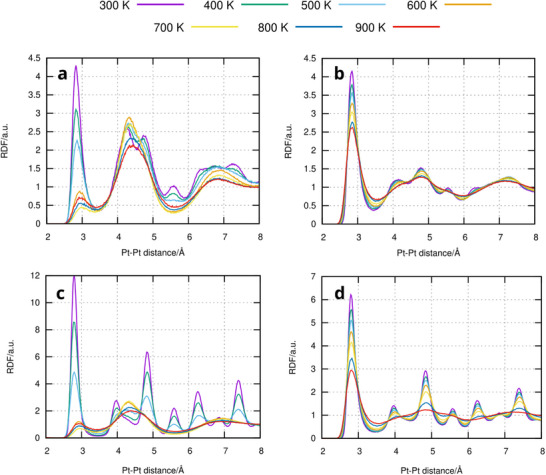
RDF plots obtained from analyzing the final 10 MD frames of the simulations for the GaPt_2_ and Pt crystals inserted into liquid GaPt with different Pt concentrations for varying temperature (see color code). a) GaPt_2_ inserted into GaPt with 16.6% Pt. b) GaPt_2_ inserted into GaPt with 50% Pt. c) Pt inserted into GaPt with 16.6% Pt. d) Pt inserted into GaPt with 50% Pt.

With **Figure** **8** the temperature‐dependence of the phase is characterized in more detail. RDF plots for systems with 16.6% and 50% Pt in the surrounding liquid containing GaPt_2_ and Pt crystals after 1.5 ns of simulations are displayed. In (a), showing GaPt_2_ in a liquid with 16.6% Pt after 1.5 ns of simulation time, the full fine‐structure can only be seen at 300 K, especially the double peak at a Pt‐Pt distance of 4–5 Å. At 400 K, the second peak has shrunk to a shoulder of the first peak. Until 500 K, the general shape stayed similar, albeit washed out. At 600 K, however, a significant change happened, especially at 3 Å, where the Pt‐Pt peak almost disappeared, indicating total melting of the crystal. In a liquid with 50% Pt content (b), no such sudden disappearance happened. The large Pt‐Pt peak at 3 Å gradually shrinks and broadens, but still stays there, the other peaks stay until 700 K and get washed out at 800 K and higher, showing that GaPt_2_ is stable up to 700 K in an environment with high Pt content.

Pure Pt again behaves like GaPt_2_. In (c), the significant peaks in the Pt‐Pt RDF stay similar and only get somewhat washed out until 500 K but suddenly switch into the characteristic RDF of liquid GaPt at 600 K, indicating the vanishing of the crystal. In a GaPt solution with 50% Pt, metallic Pt particles are stable up to 700 K, with the characteristic peaks disappearing at higher temperatures, where melting has occurred. The behavior of both particles in GaPt solution of other Pt concentrations is shown in the supporting information.

The general trend is thus confirmed: Higher Pt content of the surrounding GaPt liquid enhances the stability of GaPt_2_ and Pt nanoparticles. The formation of GaPt_2_ from Pt, which can be seen experimentally in PDF (see Figure 4), could therefore be confirmed by our MD simulations, as well as its subsequent stability, when enough dissolved Pt acts as a “protection layer” around the crystal, which could be happen in reality by a diffusion‐controlled process, where the Pt atoms dissolved from Pt nanocrystals and stay in their environment after the Pt has transformed to GaPt_2_ by gradual intercalation of Ga atoms in the crystal structure. The stability of large crystals, as approximated by the 2D periodic Pt(111) surface in the upper part (Figure 5), will be even larger than that of small ones.

It should be noted that our machine‐learned force field simulations are limited with respect to the relevant time and length scales that can be covered. We simulated time scales of up to several ns and spatial regions of some nm, such that possible effects taking place at longer time and length scales cannot be treated, for example slower IMC formation processes or influences of larger‐scale metal concentration fluctuations. Due to the averaging in the evaluation, very short‐lived (some ps) crystalline intermediates might also be neglected in the resulting RDFs. Since the MD simulations focus on much shorter time intervals than the experiments, however, the short‐time resolution of them should be better than in the experiment. The good agreement of the simulated RDFs to the experiment further suggests that no important IMC phases which could be formed at longer time scales are omitted in the simulations. The IMCs treated in the simulations are therefore indeed the dominating ones even at long time scales under the particular conditions investigated in this study.

## 4 Conclusion

Results of the PDF analysis give insight into the structure of the SCALMS catalysts before and after catalysis. Before catalysis the Ga phase is significantly oxidized to an amorphous β‐Ga_2_O_3_ phase, a process that may be influenced by sample preparation for PDF analysis. The PDF data can therefore not provide clear evidence for the existence of a residual liquid Ga core, which would confirm the often‐proposed Ga SCALMS egg‐shell model. An enrichment of Pt in the surface oxide layer can neither be confirmed nor disproved by PDF data. The oxide layer is most likely completely removed during catalyst activation, since it would otherwise lead to passivation of the catalyst's surface.

The presence of Pt_fcc_ nanoparticles can be proven by refinements for all samples prior to catalysis. The size of the nanoparticles varies with the Ga/Pt ratio, increasing from 2.5 nm in the case of Ga_177_Pt and Ga_45_Pt to 4 nm for Ga_14_Pt and Ga_8_Pt. Following catalysis, the Pt nanoparticles are fully converted into a GaPt_2_ alloy phase, which exhibits a high degree of structural disorder, which can be identified by the overall appearance of the dPDF. Notably, the GaPt_2_ alloy phase is absent in Ga_177_Pt, representing the only exception.

The ML‐FF simulations could show that GaPt_2_, being structurally similar to pure Pt, is formed at the interface between Pt(111) and liquid Ga by gradual intercalation of Ga atoms into the Pt crystal structure. The presence of this intermetallic compound with high Pt content, surprising when just looking at the overall Pt contents of the different investigated SCALMS, is thus a consequence of the preparation method and the present Pt nanoparticles. Simulations further have shown that both fcc‐Pt and GaPt_2_ are stable at temperatures up to 800 K if the Pt content of the surrounding liquid is high enough, probably due to hindered diffusion in the regions of high Pt content. It should be noted, however, that the ML‐FF simulations presented in this study do not contain the oxide layer, which seems to be of high importance in experimental measurements. Adding another element, however, greatly complicates the ML‐FF training, since the available coordinate spaces expands considerably. We are currently working to parametrize stable and reliable ML‐FFs for these composite systems, this work will be published soon.

## Conflict of Interest

The authors declare no conflict of interest.

## Author Contributions

F.E. performed investigation, formal analysis, validation; A.M. performed investigation, formal analysis, validation; J.S. performed conceptualization, wrote and reviewed; N.T. performed conceptualization, wrote and reviewed; N.R. performed investigation, formal analysis; M.Z. performed conceptualization, wrote and reviewed; A.G. performed conceptualization, supervision, funding acquisition; P.W. performed funding acquisition; M.H. performed conceptualization, supervision.

## Supporting information



Supporting Information

## Data Availability

The data that support the findings of this study are available from the corresponding author upon reasonable request.

## References

[1] P. Anastas , N. Eghbali , Chem. Soc. Rev. 2010, 39, 301. 20023854 10.1039/b918763b

[2] T. Wataria , K. Nansaia , K. Nakajima , Resour. Conserv. Recycl. 2020, 155, 104669.

[3] L. Liu , A. Corma , Chem. Rev. 2018, 118, 4981. 29658707 10.1021/acs.chemrev.7b00776 PMC6061779

[4] X. Shi , X. Lin , R. Luo , S. Wu , L. Li , Z.‐J. Zhao , J. Gong , JACS Au 2021, 1, 2100. 34977883 10.1021/jacsau.1c00355 PMC8715484

[5] O. Mohan , T. S. Choksi , A. A. Lapkin , Catal. Sci. Technol. 2024, 14, 515.

[6] N. Taccardi , M. Grabau , J. Debuschewitz , M. Distaso , M. Brandl , R. Hock , F. Maier , C. Papp , J. Erhard , C. Neiss , W. Peukert , A. Görling , H.‐P. Steinrück , P. Wasserscheid , Nat. Chem. 2017, 9, 862. 28837180 10.1038/nchem.2822

[7] N. Raman , S. Maisel , M. Grabau , N. Taccardi , J. Debuschewitz , M. Wolf , H. Wittkämper , T. Bauer , M. Wu , M. Haumann , C. Papp , A. Görling , E. Spiecker , J. Libuda , H.‐P. Steinrück , P. Wasserscheid , ACS Catal. 2019, 9, 9499. 32219008 10.1021/acscatal.9b02459 PMC7088128

[8] a) K. Zuraiqi , A. Zavabeti , F.‐M. Allioux , J. Tang , C. K. Nguyen , P. Tafazolymotie , M. Mayyas , A. V. Ramarao , M. Spencer , K. Shah , C. F. McConville , K. Kalantar‐Zadeh , K. Chiang , T. Daeneke , Joule 2020, 4, 2290;

[9] a) X. Sun , H. Li , RSC Adv. 2022, 12, 24946; 36199892 10.1039/d2ra04795k PMC9434383

[10] a) S. Zhang , R. Wang , X. Zhang , H. Zhao , RSC Adv. 2024, 14, 3936; 38288153 10.1039/d3ra07029h PMC10823358

[11] a) N. Raman , M. Wolf , M. Heller , N. Heene‐Würl , N. Taccardi , M. Haumann , P. Felfer , P. Wasserscheid , ACS Catal. 2021, 11, 13423; 34777909 10.1021/acscatal.1c01924 PMC8576810

[12] D. Esrafilzadeh , A. Zavabeti , R. Jalili , P. Atkin , J. Choi , B. J. Carey , R. Brkljača , A. P. O'Mullane , M. D. Dickey , D. L. Officer , D. R. MacFarlane , T. Daeneke , K. Kalantar‐Zadeh , Nat. Commun. 2019, 10, 865. 30808867 10.1038/s41467-019-08824-8 PMC6391491

[13] S. Carl , J. Will , N. Madubuko , A. Götz , T. Przybilla , M. Wu , N. Raman , J. Wirth , N. Taccardi , B. Apeleo Zubiri , M. Haumann , P. Wasserscheid , E. Spiecker , J. Phys. Chem. Lett. 2024, 15, 4711. 38657124 10.1021/acs.jpclett.3c03494

[14] a) T. Zimmermann , N. Madubuko , P. Groppe , T. Raczka , N. Dünninger , N. Taccardi , S. Carl , B. A. Zubiri , E. Spiecker , P. Wasserscheid , K. Mandel , M. Haumann , S. Wintzheimer , Mater. Horiz. 2023, 10, 4960; 37610262 10.1039/d3mh01020a PMC10615327

[15] a) (Ed: B.E. Warre ), in X‐Ray Diffraction, Dover Publications Inc, New York 2003;

[16] C. J. Benmore , Int. Scholarly Res. Notices 2012, 2012, 852905.

[17] S. J. L. Billinge , M. G. Kanatzidis , Chem. Commun. 2004, 749. 10.1039/b309577k 15045050

[18] S. J. L. Billinge , Int. Tables Crystallograph. 2019, 7, 649.

[19] D. A. Keen , A. L. Goodwin , M. G. Tucker , M. T. Dove , J. S. O. Evans , W. A. Crichton , M. Brunelli , Phys. Rev. Lett. 2007, 98, 225501. 17677855 10.1103/PhysRevLett.98.225501

[20] T. Egami , S. J. L. Billinge , Structural Analysis of Complex Materials, Pergamon, Oxford 2012.

[21] T.‐E. Hsieh , S. Maisel , H. Wittkämper , J. Frisch , J. Steffen , R. G. Wilks , C. Papp , A. Görling , M. Bär , J. Phys. Chem. C 2023, 127, 20484.

[22] X. Zhi , F. Xie , A. Zavabeti , G. K. Li , D. J. E. Harvie , E. Kashi , R. J. Batterham , J. Z. Liu , Energy Fuels 2023, 37, 17875.

[23] D. K. Belashchenko , Phys. Usp. 2013, 56, 1176.

[24] T. Wonglakhon , S. Maisel , A. Görling , D. Zahn , J. Chem. Phys. 2021, 154, 014109. 33412884 10.1063/5.0031185

[25] R. Jinnouchi , J. Lahnsteiner , F. Karsai , G. Kresse , M. Bokdam , Phys. Rev. Lett. 2019, 122, 225701. 31283285 10.1103/PhysRevLett.122.225701

[26] R. Jinnouchi , F. Karsai , G. Kresse , Phys. Rev. B 2019, 100, 014105. 10.1103/PhysRevLett.122.225701 31283285

[27] R. Jinnouchi , F. Karsai , C. Verdi , R. Asahi , G. Kresse , J. Chem. Phys. 2020, 152, 234102. 32571051 10.1063/5.0009491

[28] V. L. Deringer , A. P. Bartók , N. Bernstein , D. M. Wilkins , M. Ceriotti , G. Csányi , Chem. Rev. 2021, 121, 10073. 34398616 10.1021/acs.chemrev.1c00022 PMC8391963

[29] A. Shahzad , F. Yang , J. Steffen , C. Neiss , A. Panchenko , K. Götz , C. Vogel , M. Weisser , J. P. Embs , W. Petry , W. Lohstroh , A. Görling , I. Goychuk , T. Unruh , J. Phys.: Condens. Matter 2024, 36, 175403. 10.1088/1361-648X/ad1e9f 38224622

[30] E. M. Freiberger , J. Steffen , N. J. Waleska‐Wellnhofer , F. Hemauer , V. Schwaab , A. Görling , H.‐P. Steinrück , C. Papp , Nanotechnology 2024, 35, 145703. 10.1088/1361-6528/ad1201 38048605

[31] J. Steffen , A. Alibakhshi , J. Chem. Phys. 2024, 161, 184116. 39535102 10.1063/5.0226314

[32] M. Moritz , S. Maisel , N. Raman , H. Wittkämper , C. Wichmann , M. Grabau , D. Kahraman , J. Steffen , N. Taccardi , A. Görling , M. Haumann , P. Wasserscheid , H.‐P. Steinrück , C. Papp , ACS Catal. 2024, 14, 6440.

[33] I. Goychuk , A. Panchenko , A. Shahzad , J. Steffen , C. Neiss , K. Götz , C. Vogel , M. Weisser , B. Hakim , J. P. Embs , J. Englhard , J. Bachmann , H. Hildebrand , N. Denisov , P. Schmuki , A. Görling , T. Unruh , Phys. Rev. B 2025, 111, 064302. 10.1088/1361-648X/ad1e9f 38224622

[34] A. Søgaard , T.‐E. Hsieh , J. Steffen , S. Carl , M. Wu , Y. R. Ramzi , S. Maisel , J. Will , A. Efimenko , M. Gorgoi , R. G. Wilks , J. Frisch , N. Taccardi , M. Haumann , E. Spiecker , A. Görling , M. Bär , P. Wasserscheid , ChemPhysChem 2025, 26, 202400651. 10.1002/cphc.202400651 PMC12091850 39996492

[35] G. Ashiotis , A. Deschildre , Z. Nawaz , J. P. Wright , D. Karkoulis , F. E. Picca , J. Kieffer , J. Appl. Cryst. 2015, 48, 510. 25844080 10.1107/S1600576715004306 PMC4379438

[36] P. Juhás , C. Farrow , X. Yang , K. Knox , S. Billinge , Acta Cryst. 2015, 71, 562. 10.1107/S2053273315014473 26522405

[37] G. Kresse , J. Furthmüller , Phys. Rev. B Condens. Matter 1996, 54, 11169. 9984901 10.1103/physrevb.54.11169

[38] G. Kresse , J. Furthmüller , Comput. Mater. Sci. 1996, 6, 15.

[39] G. Kresse , D. Joubert , Phys. Rev. B 1999, 59, 1758.

[40] J. P. Perdew , K. Burke , M. Ernzerhof , Phys. Rev. Lett. 1996, 77, 3865. 10062328 10.1103/PhysRevLett.77.3865

[41] M. Methfessel , A. T. Paxton , Phys. Rev. B 1989, 40, 3616. 10.1103/physrevb.40.3616 9992329

[42] H. Pu , S. Grimme , J. Antony , S. Ehrlich , H. Krieg , J. Chem. Phys. 2010, 132, 154104. 20423165 10.1063/1.3382344

[43] S. Grimme , S. Ehrlich , L. Goerigk , J. Comput. Chem. 2011, 32, 21759. 10.1002/jcc.21759 21370243

[44] R. Jinnouchi , J. Lahnsteiner , F. Karsai , G. Kresse , M. Bokdam , Phys. Rev. Lett. 2019, 122, 225701. 31283285 10.1103/PhysRevLett.122.225701

[45] J. Chem. Phys. 1984, 81, 511.

[46] W. G. Hoover , Phys. Rev. A 1985, 31, 1695. 10.1103/physreva.31.1695 9895674

[47] H. Okamoto , M. E. Schlesinger , E. M. Mullerer , Ga (Gallium) Binary Alloy Phase Diagrams, ASM International, OH USA 2016, Vol. 3.

[48] V. Heine , J. Phys. C: Solid State Phys. 1968, 1, 222.

[49] J. Yang , J. S. Tse , T. Iitaka , J. Chem. Phys. 2011, 135, 044507. 21806138 10.1063/1.3615936

[50] R. Li , L. Wang , L. Li , T. Yu , H. Zhao , K. W. Chapman , Y. Wang , M. L. Rivers , P. J. Chupas , H. Mao , H. Liu , Sci. Rep. 2017, 7, 5666. 28720773 10.1038/s41598-017-05985-8 PMC5515953

[51] L. Bosio , J. Chem. Phys. 1978, 68, 1221.

[52] S. Jeller , J. Chem. Phys. 1960, 33, 676.

[53] S. J. L. Billinge , K. M. Ø. Jensen ), Atomic Pair Distribution Function Analysis, Oxford University Press, Oxford 2023, 10.1093/oso/9780198885801.001.0001.

[54] M. J. Regan , H. Tostmann , P. S. Pershan , O. M. Magnussen , E. DiMasi , B. M. Ocko , M. Deutsch , Phys. Rev. B 1997, 55, 10786.

[55] M. Grabau , S. Krick Calderón , F. Rietzler , I. Niedermaier , N. Taccardi , P. Wasserscheid , F. Maier , H.‐P. Steinrück , C. Papp , Surf. Sci. 2016, 651, 16.

[56] S. Nair , N. Coca‐Lopez , N. Madubuko , R. Portela , N. Taccardi , M. Haumann , M. A. Bañares , P. Wasserscheid , ChemCatChem 2025, 17, 202500176.

