# Supplementary material for: Supported Catalytically Active Liquid Metal Solutions (SCALMS) for Propane Dehydrogenation–Intermetallic Phases and Liquid Alloys Studied by Pair Distribution Function Analysis and Density Functional Theory
Source: Adv Sci (Weinh). 2025 Sep 9;12(44):e11498. doi: 10.1002/advs.202511498 (PMC12667473; doi:10.1002/advs.202511498)
Supplement: Supplementary file 1 — Supporting Information [file ADVS-12-e11498-s001.docx]

Supporting Information

Supported catalytically active liquid metal solutions (SCALMS) for propane dehydrogenation – intermetallic phases and liquid alloys studied by pair distribution function analysis and density functional theory

Felix Egger ^1^, Andreas Mölkner ^2^, Julien Steffen ^2^, Nicola Taccardi ^3^, Narayanan Raman ^3^, Mirijam Zobel ^1,4^*, Andreas Görling ^2,5^*, Peter Wasserscheid ^3,6^, Marco Haumann ^3,7^*

1 RWTH Aachen University, Institut für Kristallographie, Jägerstraße 17-19, 52066 Aachen, Germany

2 Friedrich-Alexander-Universität Erlangen-Nürnberg (FAU), Department Chemie und Pharmazie, Lehrstuhl für Theoretische Chemie, Egerlandstr. 3, 91058 Erlangen, Germany

3 Friedrich-Alexander-Universität Erlangen-Nürnberg (FAU), Department Chemie- und Bioingenieurwesen, Lehrstuhl für Chemische Reaktionstechnik (CRT), Egerlandstr. 3, 91058 Erlangen, Germany

4 JCNS-3: Neutron Analytics for Energy Research, Forschungszentrum Jülich GmbH, Wilhelm-Johnen-Straße, 52428 Jülich, Germany

5 Erlangen National High Performance Computing Center (NHR@FAU), Martensstr. 1, D-91058 Erlangen, Germany

6 Forschungszentrum Jülich GmbH, Helmholtz-Institut Erlangen-Nürnberg for Renewable Energy, Cauerstraße 1, 91058 Erlangen, and Institute for a Sustainable Hydrogen Ecomony (INW), Wilhelm-Johnen-Straße, 52428 Jülich, Germany

7 Research Centre for Synthesis and Catalysis, Department of Chemistry, University of Johannesburg, P.O. Box 524, Auckland Park 2006, South Africa

Corresponding authors: Mirijam Zobel [zobel@ifk.rwth-aachen.de](mailto:zobel@ifk.rwth-aachen.de); Andreas Görling [andreas.goerling@fau.de](mailto:andreas.goerling@fau.de); Marco Haumann [marco.haumann@fau.de](mailto:marco.haumann@fau.de)

# SCALMS synthesis procedure

The (Et)_3_NGaH_3_ compound was synthesized according to an established procedure [1], utilizing triethylammonium chloride (Sigma-Aldrich) as a substitute for trimethylammonium chloride. The compound was not isolated and was retained in its ethereal solution state. The gallium (Ga) content of this solution was quantified as follows: 1 mL of the ethereal solution was introduced into 10 mL of approximately 2M hydrochloric acid (HCl), and the mixture was subjected to brief boiling to facilitate the evaporation of diethyl ether. The resulting homogeneous solution was subsequently diluted to a final volume of 500 mL, and the Ga concentration was determined using inductively coupled plasma atomic emission spectroscopy (ICP-AES).

Figure S1. Schematic representation of the workflow for SCALMS preparation using the impregnation and decomposition of a gallane complex on the support material followed by galvanic displacement.

50.20 g of SiO_2_-60 (35-60 mesh) was suspended in 130 mL of dry diethyl ether under an argon atmosphere. An ethereal solution of (Et)_3_NGaH_3_ was added to this suspension in an amount calculated to achieve the target theoretical Ga loading (2 – 10 wt.%) relative to the support. The diethyl ether was removed under vacuum at approximately 243 K. After the complete evaporation of the solvent, the flask was rapidly heated to 573 K at a rate of about 10 K min^-1^ and maintained at this temperature under an argon flow. Once the decomposition of the gallane complex was complete, the resulting grey solid was kept at this temperature under a vacuum of 1 hPa overnight before being cooled and stored under an argon atmosphere.

Figure S2. Photograph of the resulting GaPt SCALMS material (a), SEM-EDS scans of the silica support (b), the Ga dispersion (c) and the Pt dispersion (d).

The relevant amount of an aqueous H_2_PtCl_6_ stock solution (4.4 mg mL^-1^ Pt) was added to 5 g of Ga-decorated SiO_2_ suspended in 30 mL of EtOH:H_2_O 5:1. After shaking for 2 hours, the materials were filtered, washed in turn with water, ethanol, and acetone. The resulting solids were dried in an oven at 403 K overnight and the Ga and Pt loadings of the prepared reference and SCALMS catalysts were determined by inductively coupled plasma atomic emission spectroscopy (ICP-AES) using a Ciros CCD (Spectro Analytical Instruments GmbH, Germany).

# SCALMS catalyzed propane dehydrogenation

Figure S3. Initial (black, P_0_) and final (red, P_20_) productivity as a function of (a) atomic ratio x in GaxPt SCALMS and (b) as a function of active metal content. Reaction conditions: 1.2 g catalyst (composition see insert), He flow 89 mL_N_ min^–1^, C_3_H_8_ flow 8.9 mL_N_ min^–1^, GHSV 4900 ml_gas_ g_Cat_._bed_^‑1^ h^-1^.

# Pair distribution function analysis


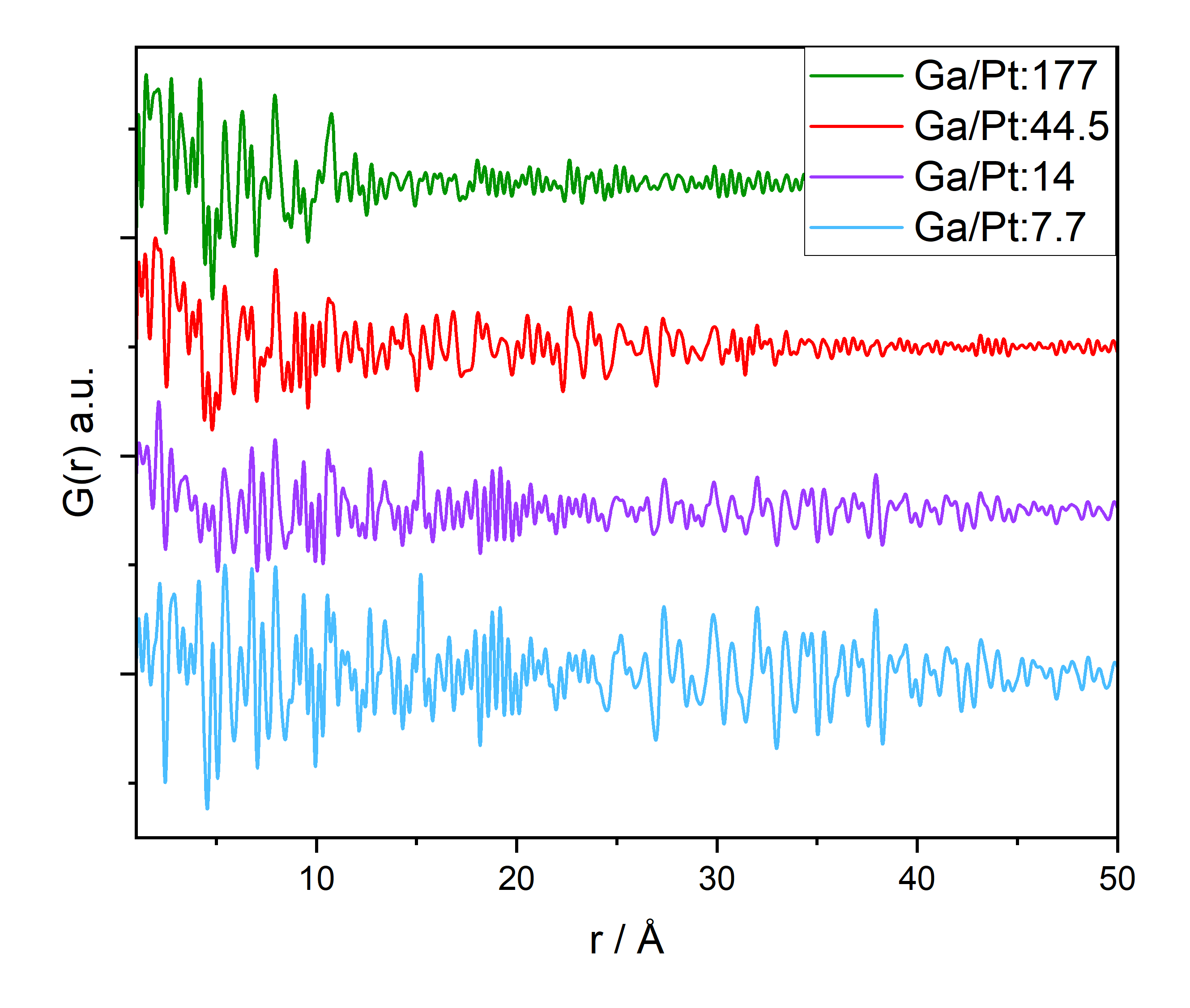


Figure S4. Difference curves of PDF refinements illustrating the residuals between the observed and calculated differential PDFs (dPDFs) for the four SCALMS catalysts. Residual structural signals primarily accumulate in the low-*r* region, indicating discrepancies in short-range structural features.

| Sample | Ga_8_Pt | Ga_8_Pt***** | Ga_14_Pt | Ga_14_Pt***** | Ga_45_Pt | Ga_45_Pt***** | Ga_177_Pt | Ga_177_Pt***** |
| --- | --- | --- | --- | --- | --- | --- | --- | --- |
| R_W_ | 0.1326 | 0.1954 | 0.1637 | 0.2303 | 0.2594 | 0.3186 | 0.2133 | 0.3118 |
| **Phase Ga_2_O_3_** |  |  |  |  |  |  |  |  |
| Ga_2_O_3_ Ga1 B_iso_ [Å^-2^] a) | 6.54E-01 | 2.71E-05 | 6.64E-01 | 5.98E-05 | 2.34E+00 | 5.18E-05 | 3.21E+00 | 9.67E-01 |
| Ga_2_O_3_ Ga2 B_iso_ [Å^-2^] | 4.54E-01 | 3.30E+00 | 4.63E-01 | 3.20E+00 | 5.65E-01 | 3.19E+00 | 5.40E-01 | 5.13E-01 |
| Ga_2_O_3_ O1 B_iso_ [Å^-2^] | 6.49E-01 | 3.75E-05 | 6.48E-01 | 3.05E-05 | 6.81E-09 | 8.56E-06 | 2.79E-06 | 3.72E-01 |
| Ga_2_O_3_ O2 B_iso_ [Å^-2^] | 6.17E-01 | 8.21E-01 | 6.10E-01 | 7.40E-01 | 5.85E-02 | 7.40E-01 | 4.20E-03 | 4.42E-01 |
| Ga_2_O_3_ O3 B_iso_ [Å^-2^] | 1.12E+00 | 5.54E-01 | 1.12E+00 | 5.48E-01 | 2.25E+00 | 5.47E-01 | 2.56E+00 | 1.11E+00 |
| Ga_2_O_3_ *delta*2 [Å^2^] b) | 3.35E+00 | 3.28E+00 | 3.34E+00 | 3.28E+00 | 3.24E+00 | 3.29E+00 | 3.11E+00 | 3.25E+00 |
| Ga_2_O_3_ *a* [Å] | 1.23E+01 | 1.27E+01 | 1.21E+01 | 1.27E+01 | 1.20E+01 | 1.27E+01 | 1.20E+01 | 1.19E+01 |
| Ga_2_O_3_ *b* [Å] | 3.05E+00 | 2.98E+00 | 3.03E+00 | 2.98E+00 | 2.99E+00 | 2.98E+00 | 2.99E+00 | 2.99E+00 |
| Ga_2_O_3_ *c* [Å] | 5.83E+00 | 5.44E+00 | 5.87E+00 | 5.44E+00 | 5.88E+00 | 5.44E+00 | 5.86E+00 | 5.82E+00 |
| Ga_2_O_3_ *β* [°] | 1.76E+00 | 1.89E+00 | 1.76E+00 | 1.89E+00 | 1.74E+00 | 1.89E+00 | 1.74E+00 | 1.76E+00 |
| Ga_2_O_3_ size [Å] | 8.59E+00 | 8.08E+00 | 7.86E+00 | 8.62E+00 | 8.18E+00 | 8.97E+00 | 8.20E+00 | 7.87E+00 |
| **Phase Pt_fcc_, refined before catalysis** | | | | |  |  |  |  |
| Pt Pt1 B_iso_ [Å^-2^] | 5.34E-01 | - c) | 4.72E-01 | - | 3.45E-01 | - | 2.76E-02 |  |
| Pt *delta*2 [Å^2^] | 3.88E+00 | - | 3.84E+00 | - | 1.27E+00 | - | 9.70E-01 |  |
| Pt *a* [Å] | 3.92E+00 | - | 3.92E+00 | - | 3.92E+00 | - | 3.92E+00 |  |
| Pt size [Å] | 3.89E+01 | - | 3.73E+01 | - | 2.50E+01 | - | 1.42E+01 |  |
| **Phase GaPt_2_ , refined after catalysis** | | | | | | | |  |
| GaPt_2_ Ga1 B_iso_ [Å^-2^] | - | 2.12E-01 | - | 2.26E-01 | - | 2.26E-01 |  |  |
| GaPt_2_ Ga2 B_iso_ [Å^-2^] | - | 9.70E-01 | - | 9.02E-01 | - | 9.02E-01 |  |  |
| GaPt_2_ Ga3 B_iso_ [Å^-2^] | - | 7.84E-01 | - | 6.59E-01 | - | 6.59E-01 |  |  |
| GaPt_2_ Pt1 B_iso_ [Å^-2^] | - | 4.83E-01 | - | 2.81E-01 | - | 2.80E-01 |  |  |
| GaPt_2_ Pt2 B_iso_ [Å^-2^] | - | 7.42E-01 | - | 6.36E-01 | - | 6.35E-01 |  |  |
| GaPt_2_ Pt3 B_iso_ [Å^-2^] | - | 1.13E+00 | - | 9.73E-01 | - | 9.71E-01 |  |  |
| GaPt_2_ Pt4 B_iso_ [Å^-2^] | - | 7.94E-01 | - | 5.81E-01 | - | 5.79E-01 |  |  |
| GaPt_2_ Pt5 B_iso_ [Å^-2^] | - | 1.25E+00 | - | 1.07E+00 | - | 1.07E+00 |  |  |
| GaPt_2_ *delta*2 [Å^2^] | - | 6.38E+00 | - | 6.66E+00 | - | 6.66E+00 |  |  |
| GaPt_2_ *a* [Å] | - | 1.63E+01 | - | 1.63E+01 | - | 1.63E+01 |  |  |
| GaPt_2_ *b* [Å] | - | 3.96E+00 | - | 3.96E+00 | - | 3.95E+00 |  |  |
| GaPt_2_ *c* [Å] | - | 5.43E+00 | - | 5.44E+00 | - | 5.44E+00 |  |  |
| GaPt_2_ size [Å] | - | 4.83E+01 | - | 4.83E+01 | - | 5.01E+01 |  |  |

Table S1. List of key refinement parameters for the four SCALMS catalysts, before and after performing propane dehydrogenation.

# Detailed description of DFT and machine learning simulations

All first-principles and machine learning (ML) calculations covered in this paper were performed with the VASP (Vienna Ab Initio Simulation Package) code, where the valence electrons are described with plane waves and the projector augmented wave (PAW) method is used to represent the atomic cores [Phys. Rev. B 199656(16), 11169-11186; Comput. Mater. Sci. 1996, 6(1), 15-50; Phys. Rev. B 199959(3), 1758-1775]. Exchange-correlation effects were described with the exchange-correlation functional developed by Perdew, Burke and Ernzerhof (PBE) [Phys. Rev. Lett.1996,77(18), 3865-3868]. An electronic cutoff of 250 eV was chosen for all DFT calculations. The convergence criterion for the electronic minimizations were always set 10^-7^ eV, in geometry optimizations, atomic positions were relaxed until a maximum force component criterion of 5*10^-3^ eV/Å was met. The energy levels at the Fermi level were broadened with the first-order Methfessel-Paxton smearing, applying a width of 0.2 eV [Phys. Rev. B1989, 40(6), 3616-3121]. Dispersion interactions were accounted for by the empirical Grimme D3 dispersion correction scheme [J. Chem. Phys. 2010, 132, 154104; J. Comput. Chem. 2011, 32, 1456]. The VASP machine-learning force field (ML-FF) based on the Gaussian approximation potential (GAP) approach [Phys. Rev. Lett.2019, 122, 225701; Phys. Rev. B2019, 100, 014105; J. Chem. Phys. 2020, 152, 234102] was used in all ML-FF simulations in this paper. In total, two ML-FFs were trained for the applications shown in this study.

For the on-the-fly learning of the ML-FF describing the dissolution of Pt atoms from a Pt surface into liquid Ga above it, the used unit cell consisted of a Pt(111) surface with 4 layers deep and 120 Pt atoms in total. On top of that 150 Ga atoms were placed on a cubic grid. All atoms were free to move. The unit cell had orthorhombic shape, with a=13.91 A, b=14.46 A and c=42 A. The reciprocal space was sampled by a 2x2x1 k-point mesh. The on-the-fly training simulation was run for 500.000 steps of 2 fs length at a constant temperature of 1500K. The Noose-Hover thermostat was used [J. Chem. Phys. 1984, 81, 511; Phys. Rev. A 1985, 31, 1695]. The maximum number of local reference configurations for the on-the-fly learning was set to 6500 while the maximum number of stored reference structures was increased automatically during the process. It was allowed for older reference configuration to be replaced by new ones. For both, the radial and the angular descriptors the cutoff was set to 8 A. For the refitting of the fast ML-FF without Bayesian error estimation the cutoffs were set to 8 and 5 A, respectively.

For the on-the-fly learning of the ML-FF describing the dissolution of GaPt_2_ and Pt crystals in GaPt SCALMS of different Pt concentrations, the training data of the first ML-FF was also taken as part of the training set. Further, a system containing 100 Ga and 100 Pt atoms was trained starting with elements placed by chance on a regular 5x5x8 grid during a linear heating of 300 to 1000 K with a time step of 2 fs for 1.000.000 steps. The maximum number of reference structures and the maximum number of local reference configurations were raised to 5500, respectively. In addition, crystalline GaPt intermetallic phases (Ga_5_Pt, Ga_7_Pt_3_, Ga_3_Pt_2_, GaPt, GaPt_2_) were learned on the fly to account for possible formations of all relevant GaPt intermetallic phases. Ga_2_Pt was not trained explicitly, since the force field trained only from the liquid GaPt (50:50) was shown to be able to crystallize Ga_2_Pt structures, which will be described in detail in a separate paper. The initial structures were built from multiples of the crystalline unit cells optimized in the first part. The GaPt_2_ simulation cell contained 64 Ga and 128 Pt atoms, the GaPt simulation cell contained 108 Ga and 108 Pt atoms, the Ga_3_Pt_2_ simulation cell contained 81 Ga and 54 Pt atoms, the Ga_7_Pt3 simulation cell contained 224 Ga and 96 Pt atoms, and the Ga_5_Pt simulation cell contained 160 Ga and 32 Pt atoms. For all four systems, reciprocal space was sampled by a 2x2x2 k-point mesh. Each system was simulated for 10.000 MD steps (2 fs time step), applying a NVT ensemble with a linear heating from 300 to 800 K. Only the GaPt_2_ system was trained at 1500 K (due to its higher melting point), with all other settings kept the same. The resulting training sets stored in ML_AB files from the in total seven independent training trajectories were finally combined with the mlff_select program from our utils4VASP repository on github [https://github.com/Trebonius91/utils4VASP].

The dissolution of a Pt(111) surface at the interface to liquid Ga was simulated with the first VASP ML-FF. The Pt(111) surface slab containing 8 layers (5x5 atoms per layer) was combined with 3 layers of liquid Ga on top of it to simulate the intrusion of Ga in the Pt surface. This cell was simulated for 24.000.000 MD steps of 2 fs length at 800 K. Further, the same cell multiplied twice along the x and y axes was simulated for 2.000.000 MD steps between 400 K and 800 K for 2 fs each.

The temperature-dependent stability of GaPt_2_ and Pt nanoparticles in liquid SCALMS of different Pt concentrations was studied at temperatures of 300 K, 400 K, 500 K, 600 K, 700 K, 800 K and 900 K with the second ML-FF. For this, cubic boxes with the liquid SCALMS were built with arbitrary Ga and Pt atoms on a cubic grid, containing 8000 atoms in total. Five different Pt concentrations were set up: 5 % Pt (7600 Ga, 400 Pt), 16.6 % Pt (6667 Ga, 1333 Pt), 25 % Pt (6000 Ga, 2000 Pt), 50 % Pt (4000 Ga, 4000 Pt), 66.6 % Pt (2667 Ga, 5333 Pt) and the cells were preequilibrated for 50.000 MD steps of 2 fs length at the respective temperatures, using the Parrinello-Rahman barostat [Phys. Rev. Lett. 1980, 45, 1196] together with the Nose-Hoover thermostat to sample the NpT ensemble with no external pressure. The inserted nanoparticles were supercells of the intermetallic phases, presampled at the corresponding temperatures, with the GaPt_2_ crystal containing 480 Ga and 960 Pt atoms and the Pt particle containing 1372 atoms. The modify_poscar.py script of the utils4VASP repository was used for insertion of the nanoparticles in the center of the liquid cells, where all atoms in the liquid with a distance lower than 2.3 A to any of the atoms in the particles were removed. Each combined cell was sampled for 300.000 MD steps of 5 fs length. For the calculation of reference radial distribution functions of the intermetallic phases, a Ga_2_Pt supercell containing 512 Ga and 1024 Pt atoms was sampled for 50.000 steps at 300 K.
